# Research on the brand image of iOS and Android smart phone operating systems based on mixed methods

**DOI:** 10.3389/fpsyg.2023.1040180

**Published:** 2023-03-06

**Authors:** Weifeng Hu, Tianyun Hao, Yue Hu, Hui Chen, Yi Zhou, Wantong Yin

**Affiliations:** School of Design, Jiangnan University, Wuxi, China

**Keywords:** iOS system, Android system, user experience, brand image, interaction behavior, mixed research methods

## Abstract

**Introduction:**

To analyze the differences in system functions, interaction behaviors and user experience between iOS and Android smart phone operating system, and then study the differences in their brand images, so as to provide theory and research method for shaping corporate brand images from the perspective of product interaction design.

**Methods:**

This study was divided into three stages. In the first stage, the functional information architecture of iOS and Android smart phone operating system are studied comparatively by using information visualization methods. In the second stage, the brand image differences between the two systems at the explicit, behavioral and semantic levels are analyzed comparatively by building the “explicit - behavioral - semantic” product brand gene model. In the third stage, the functions of “setting alarm clock”, “sharing pictures” and “modifying passwords” were selected for interactive behavior analysis. First, analyze the user experience of the three system functions from the perspective of interaction process and information architecture, and present the analysis results using the method of information visualization.; Secondly, the user experience and brand image differences between the two systems are analyzed by setting up manipulation task experiments.

**Results:**

The brand images of iOS and Android systems are similar in conciseness, clearness and efficiency; In terms of uniqueness, iOS system is more unique, while Android system has stronger applicability.

**Discussion:**

This study constructs an “explicit-behavior-semantic” brand gene model to create a unique product brand image for software products such as operating systems through interactive design, so as to solve the problem of product brand image homogeneity caused by the convergence of function and interaction design.

## 1. Introduction

Brand image is the cognitive subject’s perceptual cognition of the brand in the thinking space after contacting the brand’s products or services, and the brand image is composed of the comprehensive impression of the brand by the consumers, which is the common cognition of the brand formed by the brand and the consumers through long-term communication.

There are two main points of view in current research on improving brand competitiveness, One point of view (Aaker et al., 1993; So et al., 2017) is that brands can improve their competitiveness by creating uniqueness and difference, and enhance brand attraction through brand reputation, brand uniqueness and unforgettable brand experience, which indirectly and positively affect brand recognition; Another view (Ben Youssef et al., 2019) holds that uniqueness is an important means of shaping brand difference (Romaniuk et al., 2007). Compared with differences, brand uniqueness can help consumers pay attention to and identify brands, reduce thinking, and quickly identify a brand in a cluttered brand and advertising environment. This study believes that there is a certain correlation between uniqueness and difference, and brands can form differences with other brands through uniqueness, so as to gain advantages in the market competition.

The ISO 9241-210 standard defines user experience as the cognitive impressions and responses of people to the products, systems or services that are used or expected to be used. From the definition of brand image and user experience, we can conclude that brand image is the result of consumers’ long-term experience with a brand, so it is of great significance to study brand image based on user experience.

As the “third movement of methodology,” mixed-methods are gaining importance in research on brand image and interaction design. At the consumer level, brand image belongs to individual subjective cognition; at the level of brand ownership, the brand needs to reach a common understanding with more consumers to achieve resonance. Therefore, when studying smart phone operating system, brand, consumer and other complex ecosystem networks, traditional conventional methods have many limitations (Chen et al., 2022b,c,d).

The brand image of smart phone operating system refers to the subjective perceptual cognition of users on the operating system and brand image under the comprehensive effect of brand marketing and actual usage. The brand image of smart phone operating system is transmitted to users through multi-channel perception system by such elements as functional architecture, interaction behavior, and interface vision and interaction dynamics.

iOS and Android systems are the highest market share in today’s smart phone market. They belong to Apple and Google, respectively, and each has its own advantages, bringing different experiences to users. The brand image of the two systems has also been studied by scholars at home and abroad. Previous studies (Ling and Van Schaik, 2002; Toms, 2002; Murray and Häubl, 2011; Zhang, 2014; Yang et al., 2015; Gui, 2017; Ötz et al., 2017) have compared and analyzed iOS and Android systems from the perspective of development strategies, pointing out that Apple pursues the concept of innovation, fashion and independence, and follows the closed platform strategy; Google, on the other hand, is a “free, democratic and open” company with an open strategy and a more dynamic ecosystem. Therefore, the brand image conveyed by iOS system is “consistency, convenience and security,” while the brand image of Android system can be summarized as “diversification, personalization and freedom.” Chen (2014) compares the differences between the closed platform mode of iOS and the open platform model of Android in terms of application effects, and believes that iOS system is more conducive to improving user viscosity, and Android system improves consumers’ freedom of choice. The existing research results mostly make a comparative study on the two systems at the technical or commercial level from the functional characteristics, development strategies, ecosystems and other aspects, while the research on the brand image embodied in the system information architecture and interaction behavior is relatively lacking.

This research uses the “mixed method” to study the interactive design, user experience and brand image of the smart phone operating system functions, to analyze the enterprise’s brand strategy formulation, and comprehensively use behavior observation, user interview, semantic difference method, psychological scale, manipulation task experiment, mathematical analysis and other methods to conduct qualitative and quantitative comparative research on the smart phone brand image. The qualitative and quantitative mixed research method can obtain more comprehensive mobile phone system user experience and brand image research data, so as to analyze users’ subjective image perception of the mobile phone system brands. It is helpful to more effectively analyze the characteristics and brand image differences of iOS and Android system interaction design.

## 2. Feature analysis of iOS and Android system based on typical system function

### 2.1. Comparative analysis of system response mechanism

The response level order of Android system: Application→ Framework→ Library→ Kernal, the graphics and image processing part related to display belongs to the library level. For iOS system, when the user touches the screen, the system will give priority to deal with the screen display, i.e., cocoa touch level, followed by media level and core services level, and finally core OS level; For the Android system, after the user touches the screen, the Android system will first activate the application, then the framework and library level, and finally the kernal level. The iOS system wakes up the corresponding levels according to the user operation sequence, and the response mode is closer to the user behavior logic. Therefore, at the level of system response mode, iOS system is more smooth than Android system, which can bring users a better experience.^1^

### 2.2. Comparative analysis of system information architecture

**Figure 1:**
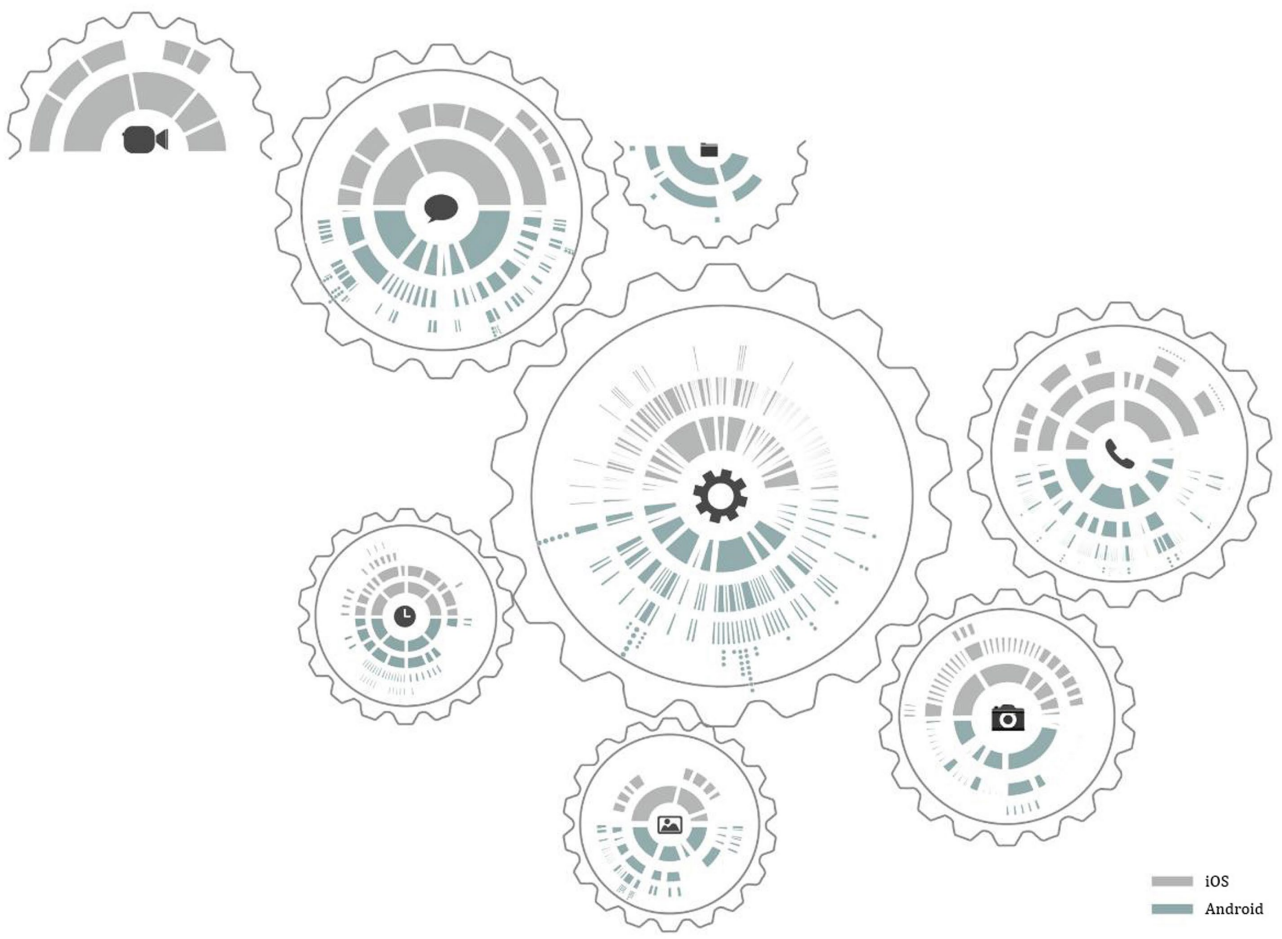
Comparative analysis of information architecture characteristics of main representative functions of iOS and Android systems (self drawn by: Lssy, Lcey, Swanna).

**Figure 2:**
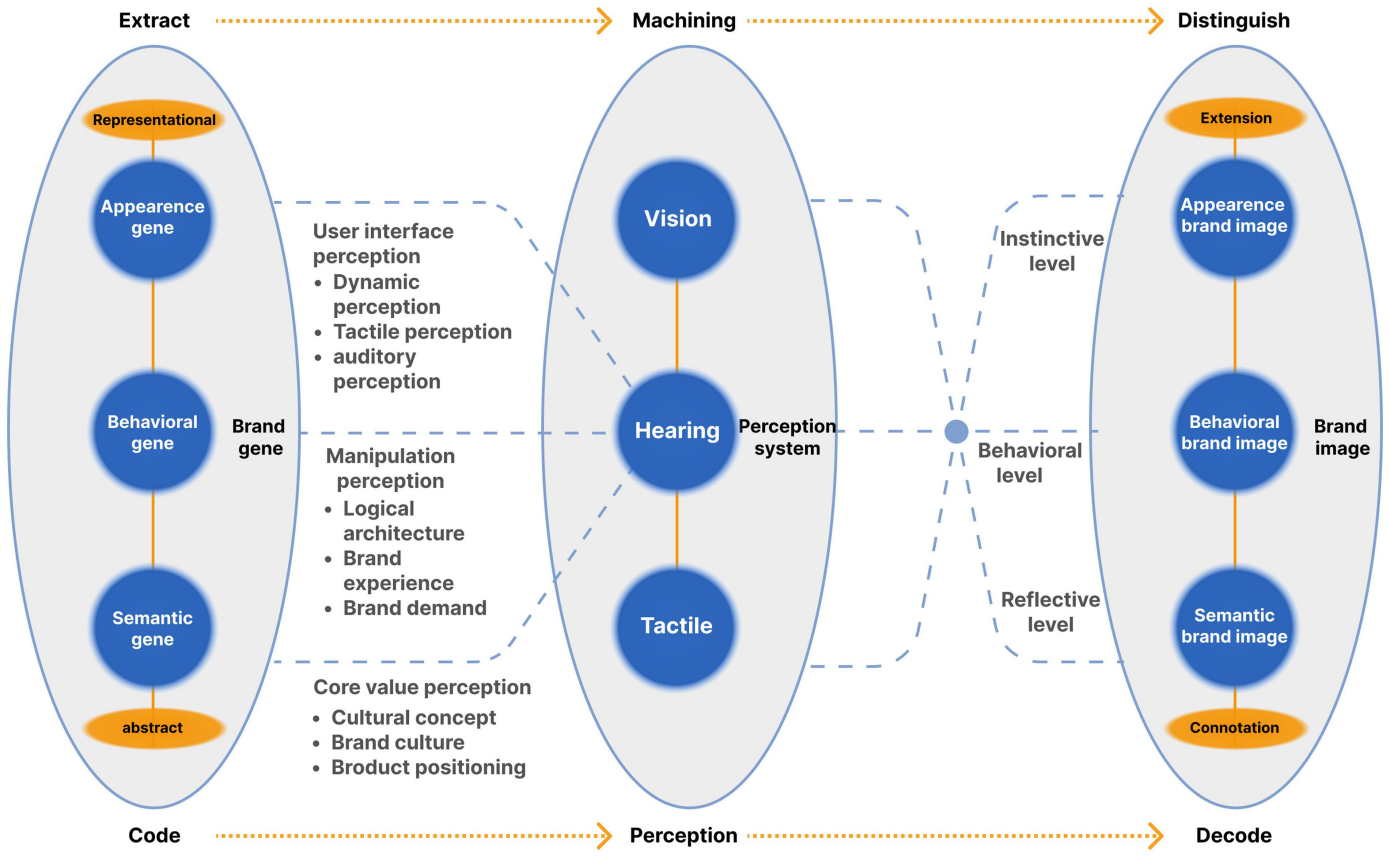
Mobile phone system brand image analysis framework.

In this paper, eight representative system functions are selected for feature analysis feature analysis: Messages, Settings, Phone, Photo, Camera, File, Clock and FaceTime, and for visual representation of the information. As shown in Figure 1, the gear shape is used as the visual expression carrier to symbolize the characteristics of coordination, unity and common operation between system’s tools. The upper half of the gear is gray, representing iOS system; The lower part is blue, representing the Android system. The first level is near the center of the gear, and the deeper the level, the farther away from the center, the number and area of color blocks of each level is determined by the number of options of this level (Figure 2).

Comparing the interaction behavior paths of some common functions of iOS and Android systems, we can find that there are significant differences between them. On the whole, the hierarchical structure of Android system is wide, which can better meet a variety of personalized needs that users may have, and provide a variety of choices and operation paths for a target function, so that users have the right to freely choose and manipulate paths, with a high degree of personalization; but, there are many hierarchies, which will lead to cumbersome operation and long operation path. The hierarchical structure of iOS system is narrow. A functional task of iOS system often provides users with limited operation ways and methods, and the operation path is usually short and the level is shallow (Hu and Xin, 2016).

**Figure 3:**
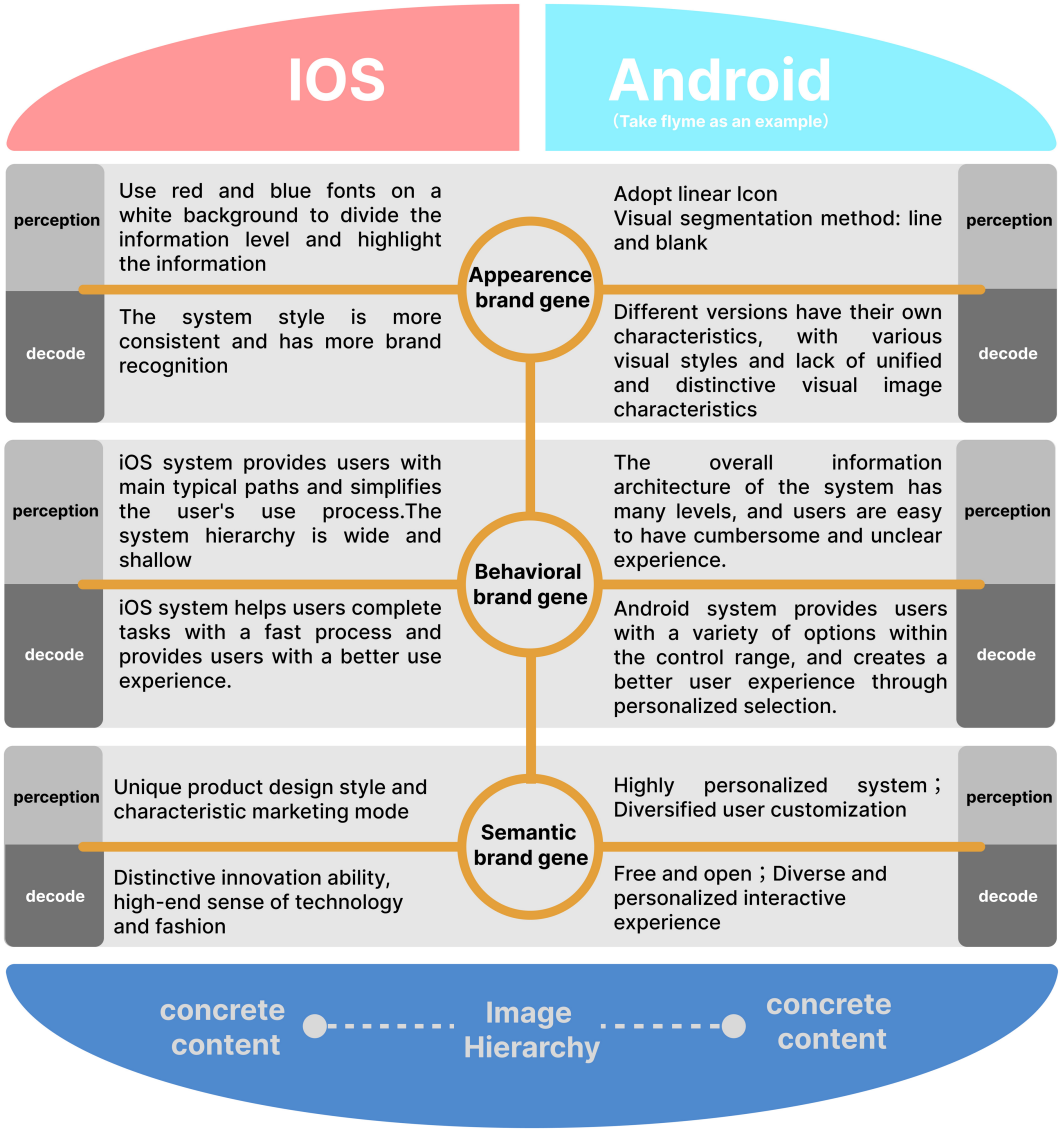
Comparison of iOS and Android brand image.

The hierarchical structure design of iOS and Android systems follows different design logics. Xin Xiangyang defines behavioral logic as “the rational organizational behavior as a basis for decision-making,” while physical logic is defined as “the basis of decision emphasizing the rational allocation of the attributes of things” (An, 2013; Xin, 2015). The main task of interaction behavior design is to be function oriented. To ensure the usability, ease of use and ease of use of products. It builds safe, comfortable, efficient, healthy and reasonable interaction behavior *via* planning the interaction mode between users and products from the time and space level (Golovanov et al., 2005; Zhou, 2016). The same is true for smart phone operating system. Based on this analysis, the hierarchical structure of iOS system pays more attention to the rationality of behavior logic, and the layout takes more consideration of function use frequency and importance (Zhang et al., 2020). The iOS system tends to make users familiar with and adapt to their given operation mode and thinking mode (Hu and Xin, 2016). It focuses on the user’s behavior and purpose, provides users with limited choices, reduces the user’s decision-making burden, and brings users a concise and efficient use experience (Figure 3).

However, the information architecture of Android system pays more attention to mathematics and classification logic, reasonably configures and classifies information according to its own attributes, pays more attention to functional relevance in layout logic, and has a complete and logically clear information architecture. Users can seek and execute functions according to their understanding of classification logic, providing more choices and meeting more personalized needs. But, when the user’s understanding of classification logic does not match the system classification logic, the user may be prone to experience confusion and difficult decision-making. Therefore, Android system pays more attention to physical logic when building information structure.

## 3. Comparative analysis of iOS and Android system brand image

**Figure 4:**
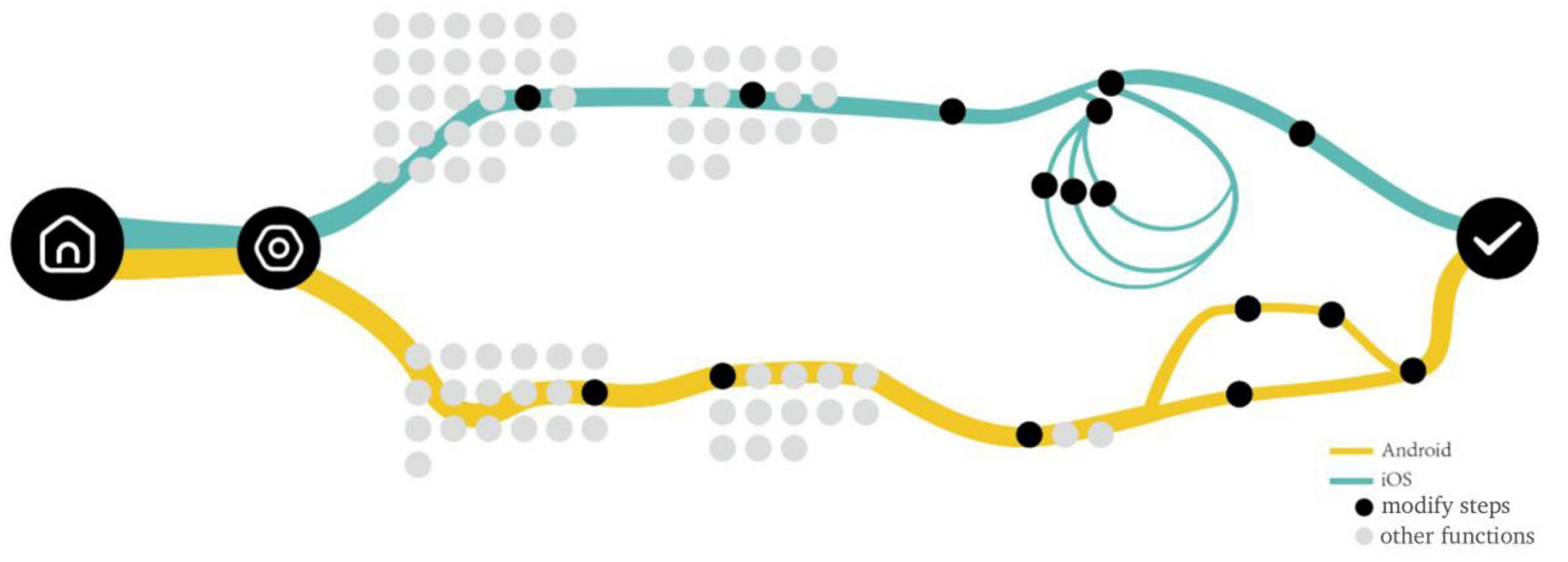
Comparison diagram of interaction flow of password modification function between iOS and Android systems (drawn by: Tianyun Hao, Yinglin Lu).

Combined with the three-level research results of “explicit gene-behavior gene-semantic gene” of software system brand gene (Yin, 2020), analyze the brand image of the two systems (as shown in Figure 4). The explicit brand gene of software system is mainly reflected in the brand image embodied by visual elements such as product color, Interface layout, interface format and text. The behavioral brand gene of software system is mainly reflected in the brand image shaped by the functional content, logical structure, manipulation behavior and other elements of the product. The semantic brand genes of the software system refer to the core values, culture and concept of enterprise brand. It is the overall value and esthetic feeling, cultural identity and impression memory generated by users after using products. It brings users a higher level of brand image in the emotional reflection layer (Yin, 2020).

### 3.1. Brand image analysis of smart phone operating system explicit gene

Explicit gene is the basic behavior of instinct layer and the formation of first impression. Perception channels such as vision, hearing and touch play a leading role in the cognition of explicit gene. Explicit gene is the most intuitive level to convey brand idea and manifest brand image, which directly affects users’ perception and experience of brand image.

In cognitive psychology, the color, shape, direction, size and spatial layout of information, and the display color combination of background and text have a significant impact on visual recognition and search efficiency (Bhattacharyya et al., 2014; Wu et al., 2021). The iOS system uses red and blue fonts on the white background to delineate information hierarchies, highlight information, direct users’ attention and improve visual search efficiency, while providing good visual recognition. However, the customized versions of the Android system are inconsistent. For example, the Android Flyme system uses gray fonts with different lightness to form differences, resulting in low visual search efficiency and visual recognition. At the “setting” level, iOS systems all use rounded rectangular icons to ensure the consistency of system icons, and use lines and negative space for information classification and visual segmentation. Negative space makes the interface concise and breathing, but too many lines may give users a sense of fatigue. The customized versions of Android system adopt different layout forms. For example, the Android Flyme system uses a linear icon. It uses white space and lines for information classification and visual segmentation. Although iOS and Android systems adopt different forms, they have achieved the effect of harmony and unity, with their own characteristics. In terms of brand image, iOS system has more consistent style and more brand recognition. Each customized version of Android system has its own characteristics, which leads to the diversity of its interface visual style and the relative lack of unified and distinctive explicit visual brand image characteristics.

### 3.2. Analysis of behavior gene and brand image of smart phone operating system

The behavior gene brand image of smart phone operating system depends on the design logic and use experience of its functional content, information architecture and interactive behavior (Huang et al., 2019) Users rarely use smart phones after fully understanding the information architecture. For the user, the function presents only a simple choice on the control path (Xin, 2015). The iOS system simplifies functions and information, and the overall hierarchical structure is relatively concise. Users will have a clear and smooth experience in the process of use. The iOS system provides users with main typical paths and simplifies the user’s use procedure. For users, they can only adapt to the way given by the system after using for a period of time. Once they form the habit of using, they will gradually improve the fluency of control, cultivate user viscosity and improve user brand loyalty (Ye et al., 2019, 2022). The overall information architecture of Android system has many levels, which will make the level of some functions too deep, making users have a relatively cumbersome and unclear experience in the search process. However, Android system takes into account the various needs of users, provides users with a variety of control paths, facilitates users to make personalized choices according to their own information needs and interaction habits, and brings users a free and humanized brand image experience.

The hierarchical structure of iOS system is wide and shallow. For example, all applications are listed at the bottom of the “Settings” page of iOS system. The hierarchical structure is wide, which is inconvenient for users to filter, increases the difficulty of finding a specific application, and brings a sense of frustration to users; To some extent, the hierarchical structure of Android system is narrow and deep. Android system places all applications under the “application management” level (the next level of “setting”), and the control path is long, which brings unclear experience to users; However, letter filtering is set on the right side of Android interface. Users can quickly find specific applications by clicking letters, bringing users a convenient use experience. Both systems have their own advantages and disadvantages. We cannot judge the advantages and disadvantages of the user experience of the two systems with a single evaluation standard. However, from the perspective of brand image, they have their own characteristics, and both have interactive behaviors with distinctive brand identity.

According to the three models of product “realization, presentation and user psychology” proposed by Cooper (2015) we can compare iOS and Android systems. Both systems are constantly optimizing the information architecture, interactive behavior and interface design to make it more in line with the user’s thinking habits and behavior, so as to narrow the distance between the presentation model and the user psychological model, but they take different ways. The iOS system helps users complete tasks with fast typical processes, providing users with a better use experience. The Android system provides users with a variety of options within the control range, and creates a better user experience through personalized selection. The iOS system helps users complete tasks with fast typical processes, providing users with a better use experience. The Android system provides users with a variety of ways to choose within the control range and create a better user experience through personalized selection. For example, Siri, the voice assistant created by iOS system, brings users a good innovative experience. Voice interaction once became the most brand recognizable interactive behavior of iOS system, but with the imitation of other brands, this brand characteristic of iOS system has been gradually weakened. With the continuous learning, improvement and optimization of the two systems, the quality of user experience has been greatly improved, but this also makes the two systems gradually converge (Fu et al., 2022). Therefore, how to build novel system manipulation and interaction behavior on the basic of good experience is the key to shaping brand differences.

### 3.3. Semantic gene brand image analysis of smart phone operating system

The semantic gene brand image of mobile phone system is closely related to the implicit elements such as the idea of corporate culture and core values (Huang et al., 2012; Chen et al., 2022a). The goal is to meet and match the emotional demands and value identity of users. Apple Inc. pursues innovative, fashionable and independent product design style and puts forward the core value concept of “making the world better.” Through its innovation capability, product quality and characteristic marketing model, Apple Inc. has enabled consumers to establish a strong sense of identity for brands and products, and created a distinctive brand image of “distinctive innovation ability, high-end sense of technology and fashion” (Chen, 2021) The iOS system adopts the closed platform strategy to maintain the consistency of user experience. The interaction behavior of system functions is concise and efficient, with a strong sense of science and technology, innovativeness, which is fully consistent with the core value of Apple Inc.’s brand. The core of Google’s innovation culture is “freedom and openness.” Google mobilized employees’ enthusiasm in various ways, created an innovative cultural atmosphere and pursued “freedom, democracy and openness.” The Android system adopts an open platform strategy, and mobile phone manufacturers can enter the high-end smart phone market without developing their own operating systems. Therefore, Android terminal devices have diverse characteristics. However, since the device manufacturer has full autonomy over the Android system, it can decide whether to update the Android system version and integrate its unique brand concept and values into the Android device design, which makes the Android system have diversified interface design styles. The opening strategy makes the Android system more energetic and diversified, but diversification also leads to the characteristic of fragmentation of the Android system.

## 4. Comparative study of user experience on iOS and Andriod system functions

This research constructs the control task experiment and user experience research of three commonly used system functions of iOS and Android, namely “setting alarm clock,” “sharing pictures” and “modifying password.” As all operations depend on the basic functions of the system, we can more realistically analyze the user experience characteristics of the two systems and summarize the brand image characteristics of the two systems.

### 4.1. Analysis of user experience on system function

#### 4.1.1. Setting alarm clock

**Figure 5:**
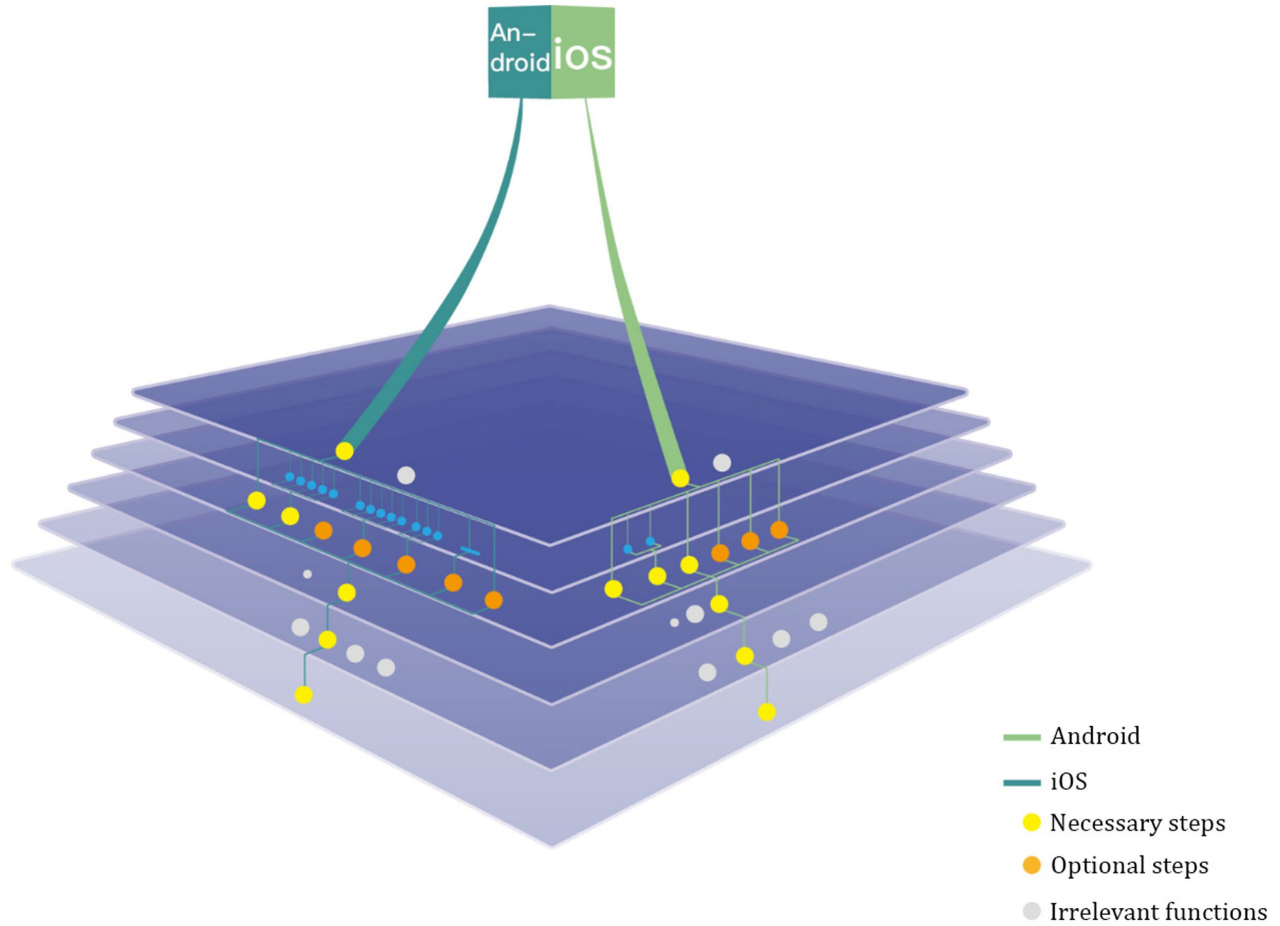
Comparison diagram of operation flow of setting alarm clock function between iOS and Android systems (drawn by: Tianyun Hao, Yichan Liu).

**Figure 6:**
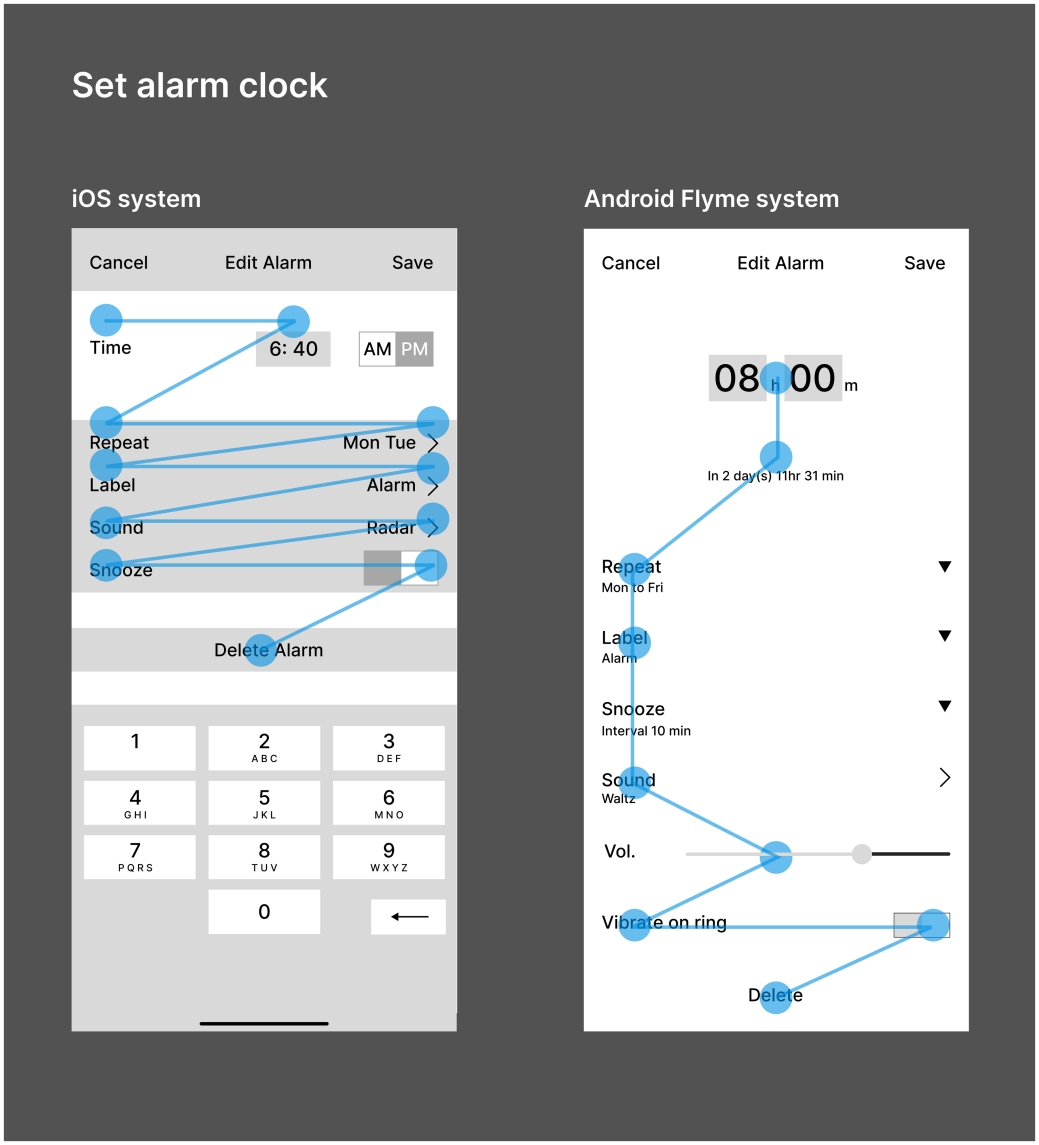
Interface diagram of setting alarm clock for IOS and Android systems (drawn by: Tianyun Hao).

“Setting alarm clock” on smart phone, the most commonly used function, is typical for comparative research. Figure 5 shows the comparison of the operation flow of setting alarm clock between iOS and Android Flyme systems. The dark green line represents the alarm setting path by Android Flyme 8.1.5.0A system, while the light green line represents the alarm setting path by iOS 14.6 system. The yellow circle represents the steps that must be taken to set the alarm clock, while the orange circle represents the steps related to but not necessary to set the alarm clock, the grey circle represents the “Cancel” button and functions unrelated to setting the alarm clock. After comparing the alarm clock setting functions of the two systems, it was found that there were the following differences:

1. Interface layout dimension. John Gutenberg proposed that people’s reading style follows the reading gravity from top left to bottom right (Lidwell et al., 2019). As shown in Figure 6, the iOS system adopts the horizontal layout of titles and options. When reading, users often turn back their line of sight, which is compact and saves vertical space, but the processing speed is slow. The titles and options of Android Flyme system are arranged up and down. When reading, users have less line of sight turn back, fast browsing and processing speed, but occupy more vertical space.
2. Information hierarchy dimension. The human cognitive system will divide the visual stimulus into two types of elements: “picture” and “bottom.” The information can be highlighted by placing it at the “picture” level (Lidwell et al., 2019). As shown in Figure 6, the iOS system emphasizes its priority by setting the time information on the level of rectangular graph, that is, the “picture”; while Android Flyme system enlarges the time number, organizes the presentation of information content through size and color comparison, emphasizes the priority of time setting, and makes users focus on time setting operation. The two systems adopt different forms of comparison, which has the effect of emphasizing priority. However, in actual use, the time setting operation of Android system has more prominent priority and larger operable area, which gives users a strong operability and error free experience.
3. Function configuration dimension. The increase of product functions can meet the personalized needs of more users, but it will also reduce the availability and ease of operation (Lidwell et al., 2019). For the implementation of alarm clock setting tasks, iOS system has few options, which brings users a concise and fast experience in some scenariOS. For example, “remind later” has no time option, which reduces the difficulty of operation. However, in other scenarios, it brings users a complex and cumbersome experience. For example, “Repeat” provides seven options from Monday to Sunday. In the scenario of setting the alarm clock on a workday, it needs to be checked five times. The interaction steps are lengthy and cumbersome; The Android Flyme system has many options, which creates a variety of experience for users. For example, “Repeat” can select five options: “Only once,” “Monday to Friday,” “Every day,” “Legal workday,” and “Custom,” which brings a humanized and efficient experience in some scenarios, but also destroys the user experience in the “Custom” scenario, To some extent, it will make the execution of tasks relatively complicated.

#### 4.1.2. Sharing pictures

**Figure 7:**
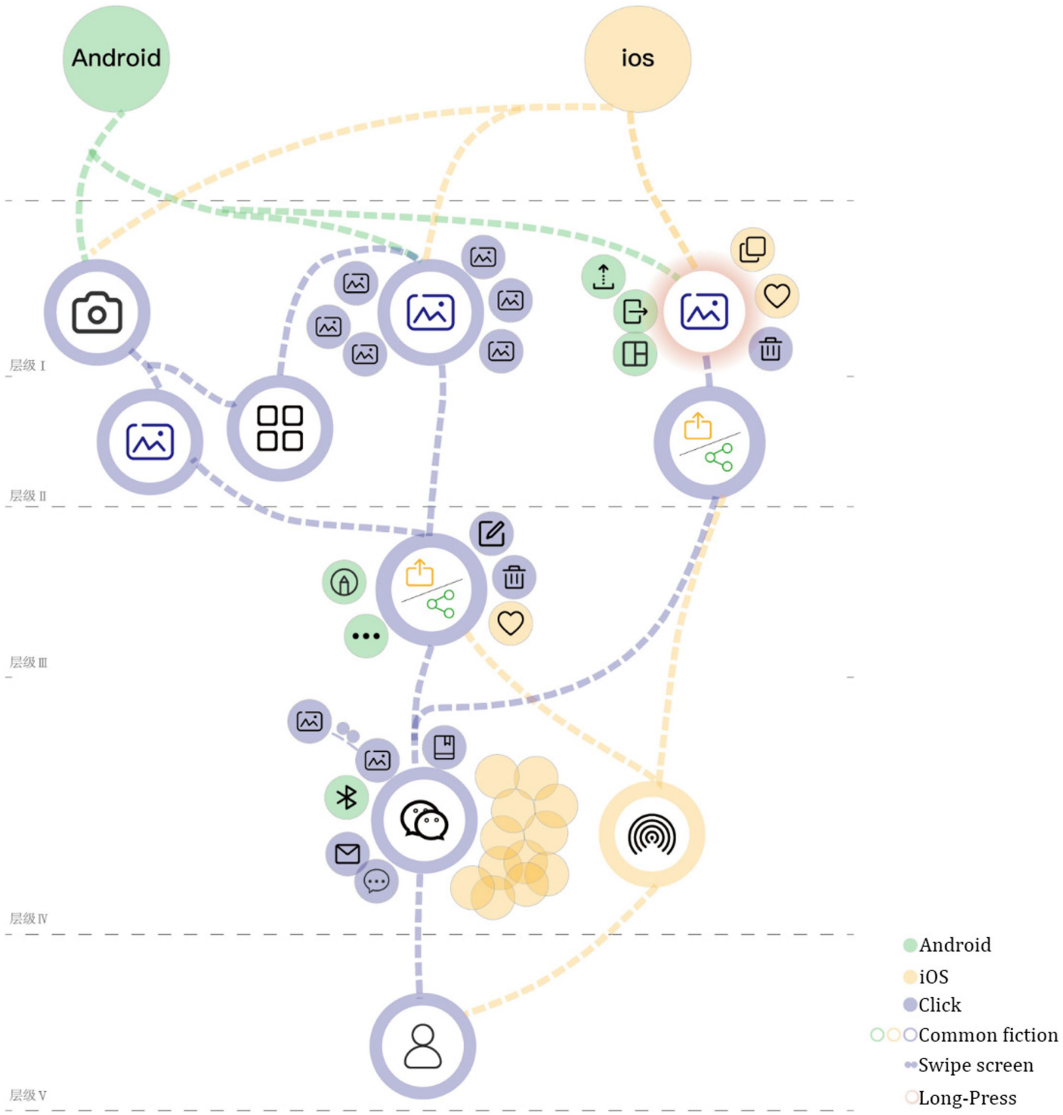
Comparison diagram of interactive process of sharing pictures between iOS and Android systems (drawn by: Tianyun Hao).

**Figure 8:**
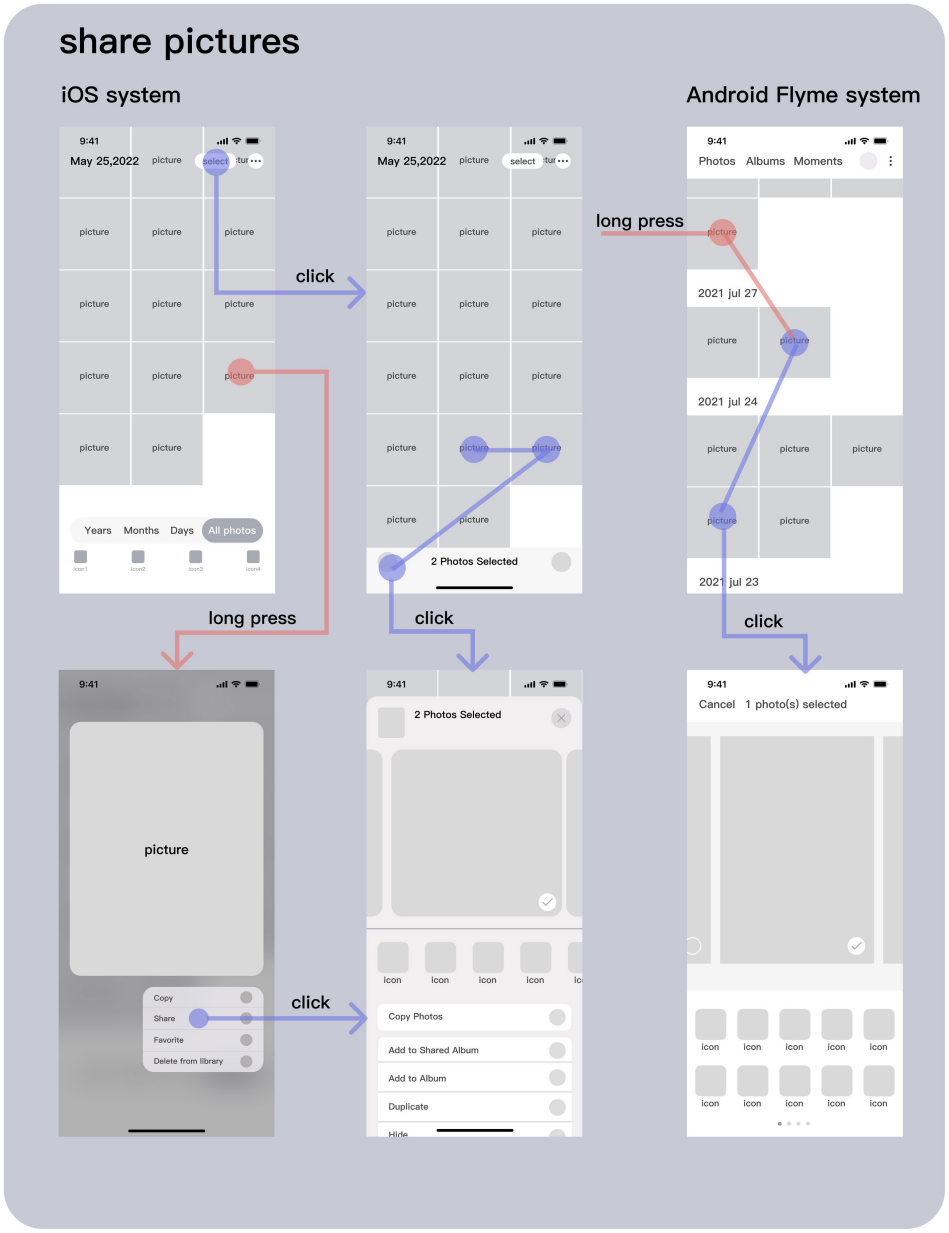
Picture sharing interface of iOS and Android system (drawn by: Tianyun Hao).

“Sharing pictures” is one of the basic system functions commonly used by mobile phones. This paper will compare the user experience of “sharing pictures” function between iOS and Android Flyme system, and summarize the differences between them. The starting points of all sharing paths of the two systems can be summarized as “camera,” “album” and “third-party software.” This paper mainly analyzes two paths starting from “camera” and “album.” As shown in Figure 7, the Android Flyme8.1.5.0A system and iOS14.6 system share a picture flow comparison diagram. The green lines and green circles in the diagram represent the paths and functions of the Android system, the yellow lines and yellow circles represent the paths and functions of the iOS system, the blue lines and blue circles represent the same functions and operations, the circle represents the “click” operation, and the red gradual change circle represents the “long press” operation. After comparing the picture sharing functions of the two systems, the following differences are found:

1. Interface layout dimension. The John Gutenberg diagram divides the information presentation layout into four quadrant areas and defines the attention effect of users on the information in each area (Lidwell et al., 2019). Fitts law (Lidwell et al., 2019) summarizes the impact of the relative distance and size of the target object on the user’s operation efficiency. Clark and Bao (2011) studies that the range of motion of the thumb has a certain impact on the operation efficiency and accuracy of the mobile phone system (Lidwell et al., 2019). According to the above research results, for the order of photos, the photos of iOS system are arranged from new to old and from bottom to top according to the shooting time, while the photos of Android Flyme system are arranged from new to old and from top to bottom according to the shooting time. Users pay high attention to the top information, but the operation is difficult. Both have their own advantages and disadvantages.
2. Operation path dimension. As shown in Figure 8, the iOS system can share pictures through “click Select - select target image - click Share” and “Long - Press target image - click Share” two ways. The “Select” button is located in a high idle area and far away from the area where users can easily operate with their thumbs, resulting in low operation efficiency and long operation path, which brings users inefficient and cumbersome experience images. Although the “long press” interactive form has a certain learning cost, the operation path is short, and in addition to sharing, the long press can also enlarge the picture, bringing users a unique experience. Android Flyme system only has a picture sharing path of “long press target picture - click to share.” Compared with the “long press” sharing path of iOS system, multiple pictures can be selected to share at the same time, bringing users an efficient use experience.
3. Hierarchy dimension. The organization and structure design of the interface is an important way to achieve concise and efficient interactive manipulation (Brown, 2010). Reasonable hierarchical structure design can help users quickly achieve their expected goals. Hierarchy is objective, which reflects the objective logic of information structure to a certain extent; However, the sense of hierarchy is subjective. The sense of hierarchy reflects the user’s understanding of the information structure. A strong sense of hierarchy will make the user have a relatively complex experience of the product, resulting in reduced user satisfaction. As shown in Figure 8, for iOS system, after clicking “Share,” the floating layer operation bar will appear based on the original scene; For Android Flyme system, after clicking “Share,” it will enter a new page for operation. Compared with Android Flyme system, the iOS system is still connected with the original scene, which weakens complexity of hierarchy and enables users to have a familiar and concise use experience.
4. Function configuration dimension. The function options provided by iOS system are concise, which does not require users to spend too much time and energy to make choices, and brings users a simple and efficient brand image experience. In addition, iOS system puts its unique sharing function “AirDrop” in the first place to facilitate users to quickly share in close-up scenes, strengthen brand image and improve users’ brand loyalty. The Android Flyme system provides many functional options, which fully consider the various needs of users and provide multiple choices. It is convenient for users to make choices according to their own needs, bringing users diversified experience, but it may also bring users operational burden.

#### 4.1.3. Modifying password

**Figure 9:**
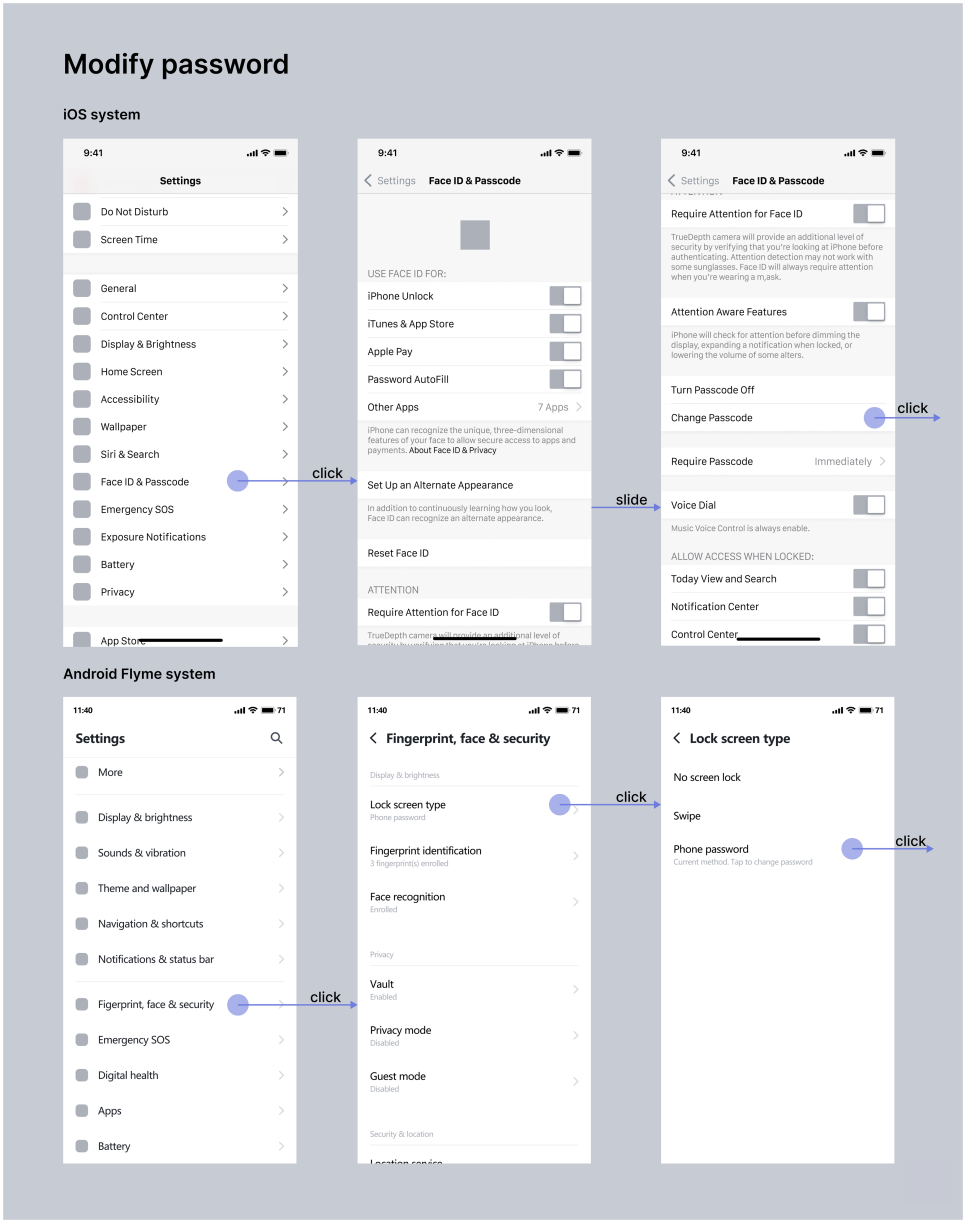
The iOS and Android system password modification interface diagram (drawn by: Tianyun Hao).

Setting and modifying password is a function that almost all users will use. Since the “setting password” task usually occurs at the stage of using a new mobile phone, the “modifying password” task is selected for comparative study in this study. As shown in Figure 4, the flow chart of password modification for iOS and Android Flyme systems is shown. The blue line represents the operating path of iOS system, the yellow line represents the operating path of Android Flyme system, the black circle represents the necessary steps for password modification, and the gray circle represents other options at the same level that are not related to password modification. After comparing the password modification functions of the two systems, it is found that there are the following differences:

1. Interface layout dimension. Proximity law is one of the representative theories of Gestalt psychology. It is proposed that the relative distance between objects can affect visual perception whether they are a whole (Liang, 2018). As shown in Figure 9, iOS system uses cards and large blank areas to group visual information, and each options in the group are divided by lines and small blank areas to strengthen the proximity of elements in the group. Compared with Android Flyme system, the information organization of iOS system is clearer and more orderly, giving users a concise visual experience.
2. Information architecture dimension. The significance of information architecture design is to show users more reasonable and meaningful information (Gullikson et al., 1999). For password modification tasks, the “Modify Password” option level of the iOS system is advanced to the “Face ID and Password” function first interface, which brings users a humanized and simple experience. The “Change Password” option of Android system is placed at a deeper level, bringing users a cumbersome and complex experience.
3. Function configuration dimension. The iOS system refines the scene, and users can customize settings to give users a strong sense of control. Android does not have such functions.

### 4.2. Manipulation task experiment

In the previous section, the interactive behaviors of the functions of “setting alarm clock,” “sharing pictures” and “modifying password” shared by the two systems were analyzed. This section will set up a manipulation task experiment to analyze the differences of user experience and brand image between the two systems.

Thirty-two subjects were selected in this experiment. In order to reduce the influence of users’ habit preference on the experimental results, about 50% of the subjects in this experiment used iOS and Android, respectively. In order to improve the accuracy of the experimental results and ensure that the subjects independently complete tasks such as “setting alarm clock,” “sharing pictures” and “modifying passwords” in the set experimental environment, the subjects are required to explain their experience in the operation process and evaluate the related brand image vocabulary after completing the tasks, so as to obtain the experimental data of brand image differences between the two systems.

With regard to the process of determining the perceptual image word pairs of smart phone system brands, firstly, 60 perceptual image words closely related to mobile phone operating system are obtained through literature research, network collection and brainstorming, as shown in Table 1. Then, an expert group composed of seven professors and graduate students majoring in design is selected to evaluate and screen the image words with their rich experience and professional knowledge combined with the functions of the mobile phone system. The differences and typicalities of image words are fully considered, ambiguous and derogatory words are eliminated, and words with similar meanings are merged. Finally, it is condensed into four image words: “Conciseness” is used to describe users’ feelings about the visual effects of the mobile phone system; “Clearness” is used to describe the user’s feelings about the operating path of the mobile phone system; “Efficiency” is used to describe users’ feelings about the operational efficiency of mobile phone systems; “Uniqueness” is used to describe the user’s experience and feelings on the comprehensive aspects of mobile phone operating system function setting, interactive behavior, interface vision, etc. Combining the three mobile phone system functions analyzed above, the brand image questionnaire is made by using the semantic difference method and the 7th-order semantic difference scale. After the subjects completed the manipulation task, they were rated for perceptual images. The scores ranged from 1 point to 7 points, with 1 point being “very different” and 7 points being “very agreed.”

**Table 1:** Collection of perceptual vocabulary of mobile phone system.

No.	Image vocabulary	No.	Image vocabulary	No.	Image vocabulary
1	Clear	21	Ingenious	41	Disjointed
2	Confused	22	Simple	42	Fuzzy
3	Consistent	23	Difficult	43	Explicit
4	Diversified	24	Elegant	44	Efficient
5	Rich	25	Chaotic	45	Inefficient
6	Delicate	26	Breathable	46	Easy to use
7	Mediocre	27	Monotonous	47	Difficult to use
8	Unique	28	Complex	48	Dense
9	Ordinary	29	Natural	49	Flexible
10	Reasonable	30	Farfetched	50	Verbose
11	Unreasonable	31	Humanized	51	Advanced
12	Clear	32	Appropriate	52	Scientific
13	Confused	33	Inappropriate	53	Pluralistic
14	Ordered	34	Unified	54	Laborious
15	Concise	35	Differentiated	55	Multifunctional
16	Complex	36	Crowded	56	Bright
17	Convenient	37	Friendly	57	Delicate
18	Cumbersome	38	Perplexing	58	Innovative
19	Meticulous	39	Single	59	Systematic
20	Coarse	40	Coherent	60	Messy

After collecting the experimental sample data, this study is divided into two stages to analyze the experimental data measured by the scale. The first stage: analyze the experimental data of brand image of iOS and Android systems, and summarize the characteristics of brand image of each system. The second stage: compare and analyze iOS and Android systems, and summarize the differences of brand image between them.

The first stage: the experimental data analysis of brand image of iOS system and Android system.

In this study, the single sample *T*-test was used to calculate the average and standard deviation of iOS system and Android system in the four dimensions of “conciseness, clearness, efficiency and uniqueness,” and the results were compared with the median value of 4. The specific conclusions are as follows.

#### 4.2.1. iOS system

1. Conciseness
  The results of single sample *T*-test of simplicity dimension of iOS system brand image are shown in Supplementary Files. iOS system conciseness dimension score mean (*M* = 5.59, SD = 1.21), significantly higher than 4, *t*(95) = 45.284, *p* <. 05, *d* = 4.62, 95%CI (5.35, 5.84).
2. Clearness
  The results of single sample *T*-test of clearness dimension of iOS system brand image are shown in Supplementary Files. The mean score of iOS clearness dimension (*M* = 5.46, SD = 1.35) was significantly higher than 4, *t*(95)=, *P* < *p* < 0.05, *d* = 4.04, 95%CI (5.18; 5.73).
3. Efficiency
  The results of single sample *T*-test of efficiency dimension of iOS system brand image are shown in Supplementary Files. The average score of iOS efficiency dimension (*M* = 5.44, SD = 1.3) was significantly higher than 4, *t*(95) = 40.845, *P* < *p* < 0.05, *d* = 4.17, 95%CI (5.17; 5.70).
4. Uniqueness
  The results of single sample T test of uniqueness dimension of iOS system brand image are shown in Supplementary Files. The average score of iOS uniqueness dimension (*M* = 4.8, SD = 1.43) was significantly higher than 4, *t*(95) = 32.568, *P* < *p* < 0.05, *d* = 3.32, 95%CI (4.46; 5.04).

Therefore, it can be considered that the brand image features embodied in the experiments of operating the functions of the three iOS systems are concise, clear, efficient and unique.

#### 4.2.2. Android system

Conciseness
  The results of single sample *T*-test of simplicity dimension of Android system brand image are shown in Supplementary Files. The average score of Android conciseness dimension (*M* = 5.34, SD = 1.38) is significantly higher than 4, *t*(95) = 37.863, *P* < *p* < 0.05, *d* = 3.86, 95%CI (5.06; 5.62).
Clearness
  The results of single sample *T*-test of clearness dimension of Android system brand image are shown in Supplementary Files. The average score of Android clearness dimension (*M* = 5.52, SD = 1.26) is significantly higher than 4, *t*(95) = 42.770, *P* < *p* < 0.05, *d* = 4.37, 95%CI (5.26; 5.78).
Efficiency
  The results of single sample *T*-test of efficiency dimension of Android system brand image are shown in Supplementary Files. The average score of Android system efficiency dimension (*M* = 5.06, SD = 1.47) is significantly higher than 4, *t*(95) = 33.715, *P* < *p* < 0.05, *d* = 3.44, 95%CI (4.76; 5.36).
Uniqueness
  The results of single sample *T*-test of uniqueness dimension of Android system brand image are shown in Supplementary Files. The average score of Android uniqueness dimension (*M* = 3.82, SD = 1.47) is significantly lower than 4, *t*(95) = 25.437, *P* < *p* < 0.05, *d* = 2.60, 95%CI (3.52; 4.12).

Therefore, it can be considered that the brand image features embodied in the three function manipulation experiments of Android system are concise, clear, efficient and not unique.

The second stage: the difference analysis of brand image between iOS and Android systems.

This paper analyzes the significant differences between the experimental data of brand image of iOS system and Android system in four dimensions: “concise, clear, efficient and unique,” and summarizes the differences of brand image between the two systems.

Firstly, the experimental data of the four dimensions are tested for homogeneity of variance. The data results are shown in Table 2. It can be found that the significance of the four dimensions based on the average is greater than 0.05, which accords with homogeneity of variance.

**Table 2:** Test of variance.

		Levin statistics	Degree of freedom 1	Degree of freedom 2	Significance
Conciseness	Based on average	2.098	1	190	0.149
	Based on median	0.870	1	190	0.352
	Based on median and with adjusted degrees of freedom	0.870	1	183.208	0.352
	Based on the average after pruning	2.006	1	190	0.158
Clearness	Based on average	0.567	1	190	0.452
	Based on median	0.071	1	190	0.790
	Based on median and with adjusted degrees of freedom	0.071	1	188.339	0.790
	Based on the average after pruning	0.431	1	190	0.512
Efficiency	Based on average	2.431	1	190	0.121
	Based on median	2.439	1	190	0.120
	Based on median and with adjusted degrees of freedom	2.439	1	185.154	0.120
	Based on the average after pruning	2.859	1	190	0.092
Uniqueness	Based on average	0.005	1	190	0.946
	Based on median	0.008	1	190	0.931
	Based on median and with adjusted degrees of freedom	0.008	1	188.572	0.931
	Based on the average after pruning	0.001	1	190	0.973

Through independent sample *T*-test, this paper analyzes the significant differences of brand images of iOS and Android in four dimensions: “conciseness, clearness, efficiency and uniqueness.” See Table 3 for the average and standard deviation of the four measurement groups, and Table 4 for the test results of independent samples.

**Table 3:** Group statistics.

	Type of system	Number of cases	Average value	Standard deviation	Mean value of standard error
Conciseness	Android	96	5.3438	1.38281	0.14113
	iOS	96	5.5938	1.21029	0.12352
Clearness	Android	96	5.5208	1.26474	0.12908
	iOS	96	5.4583	1.35271	0.13806
Efficiency	Android	96	5.0625	1.47122	0.15016
	iOS	96	5.4375	1.30434	0.13312
Uniqueness	Android	96	3.8229	1.47252	0.15029
	iOS	96	4.7500	1.42902	0.14585

**Table 4:** Independent sample test.

		Levin’s test of variance equivalence		T-test of mean equivalence						
		F	Significance	t	freedom	Significance (double tail)	Mean value difference	Standard error difference	95% confidence interval of difference	
									lower limit	upper limit
Conciseness	Assumed equal variance	2.098	0.149	−1.333	190	0.184	−0.25000	0.18755	−0.61996	0.11996
	Do not assume equal variance			−1.333	186.723	0.184	−0.25000	0.18755	−0.62000	0.12000
Clearness	Assumed equal variance	0.567	0.452	0.331	190	0.741	0.06250	0.18900	−0.31032	0.43532
	Do not assume equal variance			0.331	189.147	0.741	0.06250	0.18900	−0.31033	0.43533
Efficiency	Assumed equal variance	2.431	0.121	−1.869	190	0.063	−0.37500	0.20067	−0.77083	0.02083
	Do not assume equal variance			−1.869	187.311	0.063	−0.37500	0.20067	−0.77086	0.02086
Uniqueness	Assumed equal variance	0.005	0.946	−4.427	190	<0.001	−0.92708	0.20942	−1.34018	−0.51399
	Do not assume equal variance			−4.427	189.829	<0.001	−0.92708	0.20942	−1.34018	−0.51399

The results of independent sample *T*-test show that there are significant differences in brand image uniqueness between iOS and Android systems. It shows that the uniqueness of Android system (*M* = 3.82, SD = 1.47) is significantly lower than that of iOS system (*M* = 4.75, SD = 1.43), *t*(190) = −4.43, *p* < 0.005, *d* = −0.64, 95% CI = (−1.33). However, there are no significant differences in conciseness, clearness and efficiency.

Based on the above data analysis, the difference of brand image between iOS system and Android system is mainly uniqueness. The uniqueness of iOS system is stronger, which is consistent with the pursuit of innovation and uniqueness of iOS system; Android system has a wider application range, which is consistent with Android system’s pursuit of openness, diversity and inclusiveness. In terms of simplicity, clearness and efficiency, the brand images of iOS system and Android system are similar, mainly because both of them are constantly upgrading, learning from each other, learning from each other’s strong points and making efforts to create a better user experience.

As for whether users will choose a certain system for a long time due to their usage habits (Kim et al., 2020), research shows that whether users will continue to choose the mobile operating system they are accustomed to using when purchasing next time mainly depends on brand loyalty and satisfaction with the innovation ability of the mobile phone operating system.

## 5. Conclusion

Based on the theory of user experience, brand genes and perceptual image, this paper analyzes the differences of user experience from the perspectives of response mechanism, information architecture and interaction behavior of iOS and Android systems. A brand gene model based on “explicit-behavior-semantic” was constructed, and the characteristics and differences of brand image between the iOS and Android systems in terms of interface visuals, functional architecture and interactive behavior, brand semantics, etc. are analyzed. First, the information architecture of iOS and Android systems is analyzed as a whole by using a mixed method combining qualitative and quantitative methods; Secondly, based on the hierarchical model of brand gene, the difference of brand image between the two systems in semantic, behavioral and explicit layers is analyzed, and then the experimental verification of manipulation behavior is carried out, which fully reflects the effectiveness and rationality of the mixed-method in the research fields of smart phone operating system interaction behavior and brand image. There are few existing research results on the analysis of product brand image from the perspective of interaction design. This paper analyzes the interactive interface, information architecture, and interaction behavior of iOS and Android systems, and summarizes the brand image characteristics and differences between the two systems, enriching the research results in the field of product brand image. In addition, this study also has the following limitations:

1. This paper only selects a few typical manipulation tasks for research, which may not fully reflect the overall brand image characteristics of the interaction between the two smart phone operating system.
2. The subjects selected for this study are young people aged 22–25 who are relatively familiar with smart phones and may not be fully representative of the differences in brand image perception of users in other age groups.
3. In the quantitative analysis of this study, although the ratio of users who are accustomed to using iOS system and Android system is controlled at close to 1:1 in order to balance the experimental data, there is still a certain degree of the influence of users’ system usage preference on the experimental results.

Therefore, how to build a more recognizable product brand genetic system and shape distinctive and unique product brand imagery from the perspective of interaction design by studying the differences between the brand image of the two smart phone operating system, so as to highlight the product differentiation, is an area worthy of attention for future related research. The results of this study will provide theoretical and methodological for user experience design and brand image building of mobile phone systems, software and other products, and help to create products with high brand recognition.

## Data availability statement

The original contributions presented in the study are included in the article/Supplementary material, further inquiries can be directed to the corresponding author.

## Ethics statement

Ethical review and approval was not required for the study on human participants in accordance with the local legislation and institutional requirements. The patients/participants provided their written informed consent to participate in this study. Written informed consent was obtained from the individual(s) for the publication of any potentially identifiable images or data included in this article.

## Author contributions

WH was responsible for designing the ideas and framework of the whole paper and writing the first draft as the corresponding author. TH participated in the construction of the brand image model and the writing of the manuscript. YH participated in the writing of the manuscript. YZ participated in the revision of the manuscript. WY participated in the construction of the brand image model. All authors participated in manuscript revision, read and approved the submitted version.

## Funding

This study is supported by the Major Project of Philosophy and Social Science Research in Colleges and Universities in Jiangsu Province (Project No. 2022SJZD118).

## Conflict of interest

The authors declare that the research was conducted in the absence of any commercial or financial relationships that could be construed as a potential conflict of interest.

## Publisher’s note

All claims expressed in this article are solely those of the authors and do not necessarily represent those of their affiliated organizations, or those of the publisher, the editors and the reviewers. Any product that may be evaluated in this article, or claim that may be made by its manufacturer, is not guaranteed or endorsed by the publisher.

## Footnotes

^1^https://developer.apple.com/library/archive/documentation/MacOSX/Conceptual/OSX_Technology_Overview/CoreServicesLayer/CoreServicesLayer.html; https://developer.android.com/guide/platform
