# Supplementary material for: Research on the brand image of iOS and Android smart phone operating systems based on mixed methods
Source: Front Psychol. 2023 Mar 6;14:1040180. doi: 10.3389/fpsyg.2023.1040180 (PMC10026599; doi:10.3389/fpsyg.2023.1040180)
Supplement: Supplementary file 1 [file Data_Sheet_1.docx]

Supplementary Table 1. Single sample statistics

|  | Number of cases | Average value | Standard deviation | Mean value of standard error |
| --- | --- | --- | --- | --- |
| conciseness of iOS system | 96 | 5.5938 | 1.21029 | .12352 |

Supplementary Table 2. Single sample test (Inspection value = 0)

|  | t | freedom | Significance (double tail) | Mean value difference | 95% confidence interval of difference | |
| --- | --- | --- | --- | --- | --- | --- |
|  |  |  |  |  | lower limit | upper limit |
| conciseness of iOS system | 45.284 | 95 | .000 | 5.59375 | 5.3485 | 5.8390 |

Supplementary Table 3. Single sample statistics

|  | Number of cases | Average value | Standard deviation | Mean value of standard error |
| --- | --- | --- | --- | --- |
| clearness of iOS system | 96 | 5.4583 | 1.35271 | .13806 |

Supplementary Table 4. Single sample test (Inspection value = 0)

|  | t | freedom | Significance (double tail) | Mean value difference | 95% confidence interval of difference | |
| --- | --- | --- | --- | --- | --- | --- |
|  |  |  |  |  | lower limit | upper limit |
| clearness of iOS system | 39.536 | 95 | .000 | 5.45833 | 5.1842 | 5.7324 |

Supplementary Table 5. Single sample statistics

|  | Number of cases | Average value | Standard deviation | Mean value of standard error |
| --- | --- | --- | --- | --- |
| efficiency of iOS system | 96 | 5.4375 | 1.30434 | .13312 |

Supplementary Table 6. Single sample test (Inspection value = 0)

|  | t | freedom | Significance (double tail) | Mean value difference | 95% confidence interval of difference | |
| --- | --- | --- | --- | --- | --- | --- |
|  |  |  |  |  | lower limit | upper limit |
| efficiency of iOS system | 40.845 | 95 | .000 | 5.43750 | 5.1732 | 5.7018 |

Supplementary Table 7. Single sample statistics

|  | Number of cases | Average value | Standard deviation | Mean value of standard error |
| --- | --- | --- | --- | --- |
| uniqueness of iOS system | 96 | 4.7500 | 1.42902 | .14585 |

Supplementary Table 8. Single sample test (Inspection value = 0)

|  | t | freedom | Significance (double tail) | Mean value difference | 95% confidence interval of difference | |
| --- | --- | --- | --- | --- | --- | --- |
|  |  |  |  |  | lower limit | upper limit |
| uniqueness of iOS system | 32.568 | 95 | .000 | 4.75000 | 4.4605 | 5.0395 |

Supplementary Table 9. Single sample statistics

|  | Number of cases | Average value | Standard deviation | Mean value of standard error |
| --- | --- | --- | --- | --- |
| conciseness of Android system | 96 | 5.3438 | 1.38281 | .14113 |

Supplementary Table 10. Single sample test (Inspection value = 0)

|  | t | freedom | Significance (double tail) | Mean value difference | 95% confidence interval of difference | |
| --- | --- | --- | --- | --- | --- | --- |
|  |  |  |  |  | lower limit | upper limit |
| conciseness of Android system | 37.863 | 95 | .000 | 5.34375 | 5.0636 | 5.6239 |

Supplementary Table 11. Single sample statistics

|  | Number of cases | Average value | Standard deviation | Mean value of standard error |
| --- | --- | --- | --- | --- |
| clearness of Android system | 96 | 5.5208 | 1.26474 | .12908 |

Supplementary Table 12. Single sample test (Inspection value = 0)

|  | t | freedom | Significance (double tail) | Mean value difference | 95% confidence interval of difference | |
| --- | --- | --- | --- | --- | --- | --- |
|  |  |  |  |  | lower limit | upper limit |
| clearness of Android system | 42.770 | 95 | .000 | 5.52083 | 5.2646 | 5.7771 |

Supplementary Table 13. Single sample statistics

|  | Number of cases | Average value | Standard deviation | Mean value of standard error |
| --- | --- | --- | --- | --- |
| efficiency of iOS system | 96 | 5.0625 | 1.47122 | .15016 |

Supplementary Table 14. Single sample test (Inspection value = 0)

|  | t | freedom | Significance (double tail) | Mean value difference | 95% confidence interval of difference | |
| --- | --- | --- | --- | --- | --- | --- |
|  |  |  |  |  | lower limit | upper limit |
| efficiency of iOS system | 33.715 | 95 | .000 | 5.06250 | 4.7644 | 5.3606 |

Supplementary Table 15. Single sample statistics

|  | Number of cases | Average value | Standard deviation | Mean value of standard error |
| --- | --- | --- | --- | --- |
| uniqueness of iOS system | 96 | 3.8229 | 1.47252 | .15029 |

Supplementary Table 16. Single sample test ( Inspection value = 0 )

|  | t | freedom | Significance (double tail) | Mean value difference | 95% confidence interval of difference | |
| --- | --- | --- | --- | --- | --- | --- |
|  |  |  |  |  | lower limit | upper limit |
| uniqueness of iOS system | 25.437 | 95 | .000 | 3.82292 | 3.5246 | 4.1213 |
